# The Mediating Role of Resilience and Self-Esteem Between Life Events and Coping Styles Among Rural Left-Behind Adolescents in China: A Cross-Sectional Study

**DOI:** 10.3389/fpsyt.2020.560556

**Published:** 2020-11-20

**Authors:** Juan Li, Yi-ping Chen, Jie Zhang, Meng-meng Lv, Maritta Välimäki, Yi-fei Li, Si-lan Yang, Ying-xiang Tao, Bi-yun Ye, Chu-xia Tan, Jing-ping Zhang

**Affiliations:** ^1^Xiangya Nursing School of Central South University, Changsha, China; ^2^Alice Lee Centre for Nursing Studies, Yong Loo Lin School of Medicine, National University of Singapore, Singapore, Singapore; ^3^Department of Nursing Science, University of Turku, Turku, Finland

**Keywords:** left-behind adolescents, life event, resilience, self-esteem, coping style

## Abstract

**Objective:** This study aims to explore the association between life events and coping styles, and how resilience and self-esteem mediate the association.

**Methods:** A cross-sectional study was conducted among 981 left-behind adolescents (LBAs) in five junior high schools in Hunan Province, China, from April 13 to April 20, 2020. We utilized self-designed sociodemographic questionnaire, Adolescent Self-Rating Life Events Checklist, Resilience Scale Chinese Adolescent, Rosenberg Self-Esteem Scale, and Simplified Coping Style Questionnaire to assess the mental health of LBAs. Statistic description, Pearson correlation analysis, and structural equation model were adopted to analyze the data.

**Results:** Results revealed that life events could negatively predict resilience (β = −0.29, *P* < 0.001) and self-esteem (β = −0.39, *P* < 0.001) and positively predict LBAs' positive coping style (β = 0.28, *P* < 0.001) and negative coping style (β = 0.21, *P* < 0.001). Self-esteem could also positively predict the resilience of LBAs (β = 0.62, *P* < 0.001); resilience could negatively predict the negative coping style (β = −0.21, *P* < 0.001) and positively predict the positive coping style (β = 0.79, *P* < 0.001). Life events not only have direct effects on negative coping style (β = 0.21) and positive coping style (β = 0.28) but also have indirect effects on coping styles by affecting resilience (β = −0.29) and self-esteem (β = −0.39). The total effect of life events on coping styles was 0.32, where 34.37% was mediated by resilience and self-esteem.

**Conclusion:** We proved that resilience and self-esteem mediated most of the effects of life events on coping styles. The findings had important implications for interventions to promote mental health of LBAs, particularly the enhancement of resilience and self-esteem.

## Introduction

The widening gap in social and economic development between China's urban and rural areas and the relaxation of immigration restrictions have led to a large number of rural laborers leaving the countryside and seeking better job opportunities in cities. Children had to separate from their parents because of financial constraints and the transient nature of the work in urban areas (1). According to the results of the latest Chinese census of population (2), the number of left-behind children who refer to the individuals aged 0–18 years with one or both parents who are migrant workers (3) has exceeded 60 million, and left-behind adolescents (LBAs) who aged 11–18 years account for 29.62% of the total number of left-behind children (4). The parent–child separation could adversely affect children's psychological and social development (5, 6). Although adolescents may be more independent than younger children, at this crucial age of growth and development, because of parental absence and lack of supervision, LBAs are more likely to have anxiety and depressive symptoms than non–left-behind adolescents (NLBAs) (7, 8), even higher prevalence of self-harm (9) and suicide attempt (10).

Life events refer to events or situations that challenge, threaten, damage, or exceed the physical and mental capacity of individuals (11). Compared to NLBAs, LBAs' living environments are more stressed (12–14), and they experience more life events because of parent–child separation. They have a smaller social circle, worse nutritional status, and more housework (15) and even need to take care of aged or sick caregivers (16). They feel a higher degree of pressure derived from life events such as interpersonal relationship, study pressure, punishment, sense of loss, and healthy adaptation (8, 12, 14, 17). According to Jiang's (18) Mental Stress System model, life events are important risk factors for adolescent development. As stressors, they cause the body to make physiological and psychological adjustments, resulting in stress reactions, changes in the secretion of body hormones (19), and significant effects on psychological and social end results (20).

Coping style is the process of managing external or internal demands and an important intermediary regulating factor in the process of psychological stress (21). Stone and Neate proposed eight coping styles, such as distraction, re-evaluation of the environment, catharsis, and relaxation (22). Lazarus and Folkman also proposed eight specific coping methods, such as confrontation, avoidance, self-control, and seeking support (23). Yang proposed six types of coping methods, such as selective neglect, changing the value system, recklessness, or taking risks (24). From the perspectives and research results of different researchers, it can be seen that the coping methods are indeed diverse; however, further analysis found that the coping methods proposed by different researchers have common characteristics, that is, some coping methods have more positive components, such as seeking support and trying to change, and some are mainly negative elements, such as avoidance and vent. The positive and negative characteristics of coping methods can indeed be observed in real life and are easy to be recognized and understood by people; therefore, Xie proposed to divide coping methods into positive coping styles and negative coping styles (25). Positive coping styles will help alleviate the impact on individuals and maintain both physical or mental health (26), whereas negative coping styles can damage mental health (27). A recent meta-analysis (28) found that when LBAs face stressful events, both positive and negative coping styles coexist; however, they adopt more negative coping styles. Compared with positive coping styles, LBAs who adopt negative coping styles have lower life satisfaction and more problems such as online game addiction. In the face of life events, if there is no positive coping style, the risk of psychological damage is more than twice than that of the general population, which can be as high as 43.3% (29). Therefore, it is necessary to have an in-depth discussion on the coping styles of LBAs, which will provide targeted guidance for LBAs to adopt active coping styles and avoid more serious psychological impacts.

Researchers found that some children develop well in some stressful situations, even beyond the level of normal children; resilience acts as an intermediary between adverse situation and good adaptation (30). Kumpfer (31) also emphasizes the importance of positive cognition in its model of resilience action mechanism. Previous studies found that resilience can attenuate the mental health problems of young adults who experienced childhood adversity (i.e., abuse, neglect, and household dysfunction in childhood and adolescence) (32, 33). Studies (34–36) have shown that there was a significant negative correlation between life events and resilience in adolescents, and coping styles were found to be significantly associated with adolescent resilience (37–39). Taken together, resilience may mediate the relationship between life events and coping styles.

Self-esteem is also an important factor affecting psychological stress response (40). The Adolescent Resilience Model (41) demonstrated that self-esteem could offset the negative effects of life events. Self-esteem is also considered an important protective factor of resilience (42) and proves to have a certain predictive effect on resilience (43–45). Prior research shown that self-esteem is a crucial individual difference variable that closely related to life events (46, 47), and higher self-esteem can even promote adolescents' positive adaptation of life events (48, 49) and reduce the risk of mental health problems (50). Self-esteem was significantly correlated with coping styles (51) among adolescents. Thus, self-esteem may not only mediate the relationship between life events and coping styles directly but also mediate the relationship *via* resilience.

Although some researchers have investigated the relationships between life events, resilience, self-esteem, and coping styles, few researches have explored the protective factors of coping styles among LBAs in China, as well as the comprehensive relationships between these variables. In particular, few studies have divided coping styles into positive and negative coping styles in-depth discussion among LBAs. Considering parent–child separation is long-standing and widespread in the context of Chinese social environment, rapid change or reduction of the life events of LBAs may not be possible, whereas resilience and self-esteem can be changed under appropriate intervention (52–54). Accordingly, we aimed to explore the relationships of life events, coping styles, resilience, and self-esteem among LBAs based on the structural equation model (SEM) (55), which is an analytical method used to analyze complex relationships and causal paths when involving potential structures, providing a reference conceptual framework for the prevention and intervention to help LBAs to cope with life events positively.

**Figure 1:**
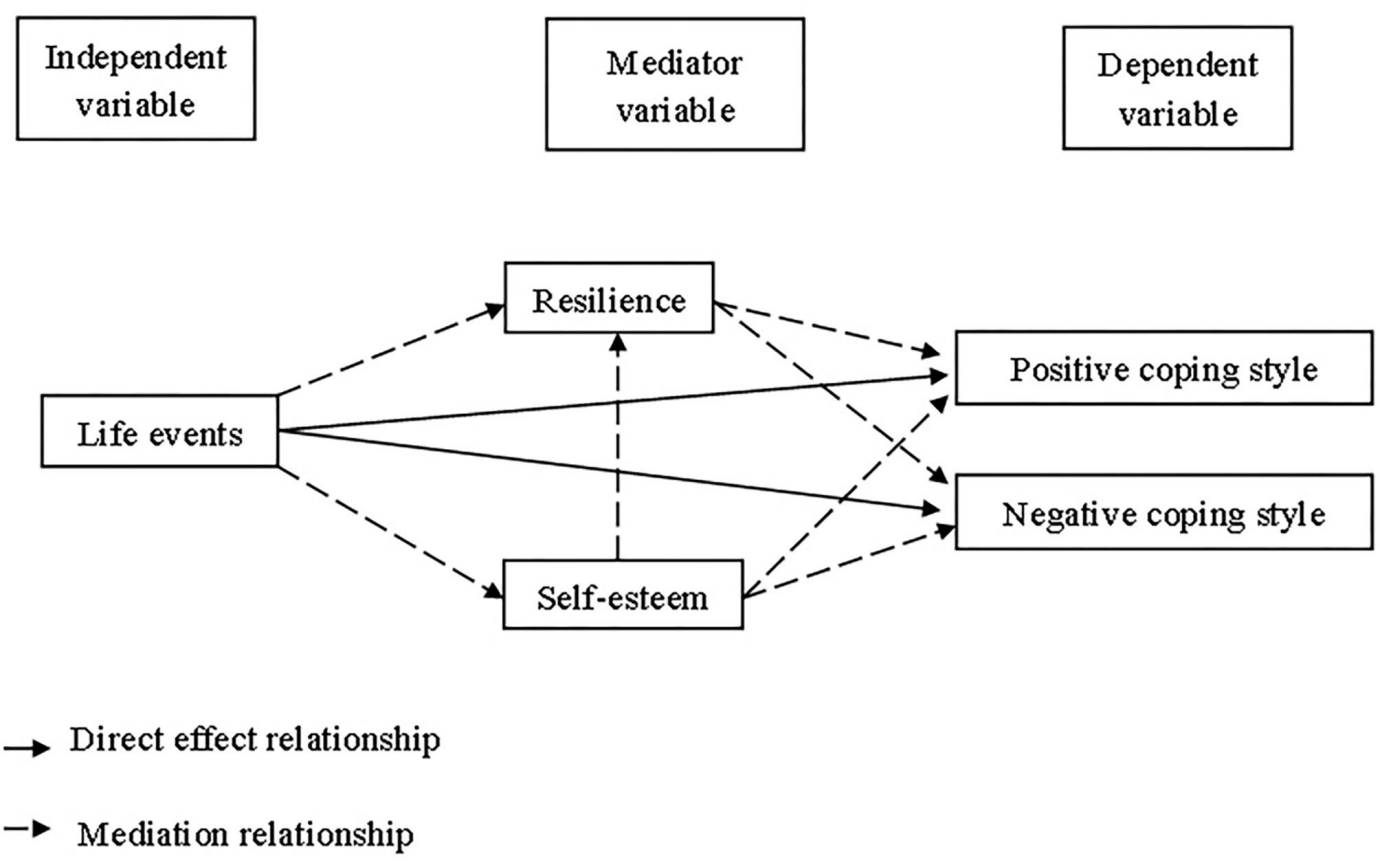
Hypothetical relationship diagram.

Based on literature review, a theoretical hypothesis model, as shown in Figure 1, was established, and we proposed the following three hypotheses: (1) life events are negatively related to positive coping styles and positively related to negative coping styles; (2) resilience mediates the relationship between life events and coping styles; and (3) self-esteem mediates the relationship between life events and coping styles *via* resilience.

## Methods

### Study Design and Sample

A cross-sectional study was conducted among LBAs in Hunan Province, China, from April 13 to April 20, 2020. The number of LBAs in Hunan Province accounts for 10.1% of the total number of LBAs in China, making it one of the provinces with the largest number of LBAs in the country (56). From Hunan province, parents migrate to work to some economically developed areas, such as Guangdong, Zhejiang, Yunnan, and other provinces (57).

The inclusion criteria for participants were as follows: (a) one or both of their parents are out-migrant workers for at least 6 months; (b) aged between 11 and 18 years; and (c) they are conscious and have volunteered for the study. Correspondingly, the exclusion criteria were as follows: (a) participants who are illiterate; (b) participants with insanity or severe mental disorders and inadequate communication ability assessed by psychiatrists; and (c) participants with hearing or speech dysfunctions.

Two-step stratified random sampling was used in the study. First, we randomly selected one city in each of five administrative regions of Hunan province. Second, one rural junior high school in each sampled city was chosen randomly. In total, five rural junior high schools were included. Before data collection, we received the permissions from the headmaster and head teacher of each school. With the help of the head teachers, we gathered the participants in several classrooms and handed out questionnaires to them without the presence of teachers. After all questionnaires were collected, we divided the participants into LBA or NLBA group according to the answer to the question, “Did one or both of your parents out-migrant for work for at least 6 months?” and selected the LBAs' questionnaires for analysis. Finally, we recruited 1,000 LBAs in the study, and 19 students refused to participate or returned incomplete questionnaires. Thus, 981 questionnaires were available, and the response rate was 97.32%.

### Sample Size

The formula of mean sampling was selected, as shown in *N* = [*U*_α_σ/δ]^2^. *U*_α_ was the value of υ corresponding to the test level α, σ was the overall standard deviation, and δ was the allowable error. Based on the preliminary experiment, the standard deviation was σ = 1.47. Taking α = 0.05, δ = 0.1, the minimum sample size was 830. Considering the 20% of the loss of access rate and sampling error, the sample size expanded to 1,000 (58).

### Measures

Sociodemographic characteristics were measured by self-designed questionnaire, including LBAs' age, gender, grade, yearly income [<10,000 RMB (poor), 10,000–20,000 RMB (intermediate), >20,000 RMB (high), and unclear], source of income, father or mother as out-migrant worker, working duration of parents, educational level of parents, occupation of parents, and contact frequency.

Life events were measured by the Adolescent Self-Rating Life Events Checklist (ASLEC) (59), which consisted of 27 items in six factors, including interpersonal relationship, study pressure, punishment, sense of loss, healthy adaptation, and other factors. The scale was used to assess the frequency and intensity of negative life events that may bring psychological effects to adolescents (60). Each item was evaluated on a six-point Likert scale, ranging from 0 (did not occur) to 5 (extremely severe), and a higher score indicated greater stress. In this study, the Cronbach values of the ASLEC scale and each subscale were between 0.78 and 0.92.

Resilience was examined by the Resilience Scale Chinese Adolescent (61). The scale comprised 27 items and two dimensions, including personal and support strength. The personal strength consisted of three factors: goal focus, emotional control, and positive perception, and the support strength consisted of two factors: family support and interpersonal assistance. Five factors reflected the effectiveness of adolescents' cognition, emotions, behaviors, and environment to help them resist adversity. Each item was scored from 1 (completely inconsistent) to 5 (completely consistent), with higher scores indicating higher level of resilience. According to the 27% delimitation principle, we sorted resilience scores among all the patients in descending order. The first 27% were known as high-level resilience, the last 27% were as low-level resilience, and then the middle part was medium-level resilience. The Cronbach value of the total scale was 0.85.

Self-esteem was determined by the Rosenberg Self-Esteem Scale (62). The scale consisted of 10 items and aimed to assess adolescents' overall feelings about self-worth and self-acceptance. Each item was evaluated on a five-point Likert scale ranging from 1 (completely inconsistent) to 5 (completely consistent), with higher scores indicating higher level of self-esteem. The scale divided an individual's self-esteem into four levels: the score of feeling inferiority was between 10 and 15 points; the average person scored between 16 and 25 points (such individuals felt ordinary about themselves); the score of feeling confident was 26–35 points (feeling better about themselves); and super-confident people who scored 36–40 are fairly confident in themselves but should also learn to be humble. The Cronbach value of the total scale was 0.82.

Coping style was measured by the Simplified Coping Style Questionnaire (SCSQ) (25). The scale comprised 20 items and two dimensions: the positive coping style dimension including 1–12 items and the negative coping style dimension including 13–20 items. Each item was ranked from 0 (never) to 4 (always). The Cronbach values of the whole scale and the two dimensions were 0.90, 0.89, and 0.78, respectively.

### Ethics Statement

The ethics approval was obtained from the ethics committee of our university (no. E201946) before data collection. The permissions to collect the data were guaranteed by the rector and head teacher of each school. Prior to filling out any questionnaires, all participants were informed about the purpose of the study and signed an informed consent form, which informed that the whole study process was carried out completely voluntarily, anonymously, and confidentially. Further, participants have the rights to decline the study at any time without any penalty.

### Data Analysis

Data were put in and analyzed using SPSS 20.0 and AMOS 23.0. First, we checked for missing values, outliers, and normality and excluded 10 missing data before data analysis. Second, summative score values for each scale were calculated, and the relationships between variables *via* Pearson correlation analysis were examined. Third, the SEM was used to determine the hypothetical mediation model, and the relationship between variables was determined by using AMOS 23.0. Specifically, a hypothetical model of coping style to life events was constructed. We set life events as exogenous explicit variables, resilience and self-esteem as endogenous explicit variables, and positive coping styles and negative coping styles as endogenous latent variables. We then estimated the path coefficient and evaluated the data fit of each model.

The goodness of fit of the model was evaluated using χ^2^ statistics [χ^2^/degrees of freedom (*df* )] and root mean square error of approximation (RMSEA). If the χ^2^/*df* was <3, the model would be regarded as a good fit; if it was between 3 and 5, the model would be considered an acceptable fit. Furthermore, if the RMSEA value was <0.05, it would indicate that the model had reached a close fit, and if the value was <0.08, the model would be counted as a good fit. Additionally, fit indices were the comparative fit index (CFI), goodness-of-fit index (GFI), normed fit index (NFI), relative fit index (RFI), and Tucker–Lewis index (TLI). If the CFI, GFI, NFI, RFI, and TLI values were 0.90 or higher, it would indicate that the model achieved a good fit (63).

## Results

### Descriptive Statistics

As shown in Table 1, participants' ages ranged from 11 to 16 years (mean = 13.48, *SD* = 0.92). Of the total participants, 51.9% were males, and 48.1% were females. Less than two-thirds of LBAs' parents were out-migrant workers (58.1%), whereas 33.2% of LBAs' fathers were out-migrant workers, and only 8.7% of LBAs' mothers were out-migrant workers. Most of the fathers migrated to work in other cities for about 6–10 years (39.0%), whereas most of the mothers migrated to work in other cities for less than a year (50.6%).

**Table 1:** The differences among sample characteristics, life events, resilience, self-esteem, and coping style (*N* = 981).

**Variable**	**N (%)**	**Life events**			**Resilience**			**Self-esteem**			**Coping style**		
		**Mean (SD)**	***t* or *F***	***P***	**Mean (SD)**	***t* or *F***	***P***	**Mean (SD)**	***t* or *F***	***P***	**Mean (SD)**	***t* or *F***	***P***
Gender			*t* = 0.844	0.399		*t* = 0.010	0.992		*t* = 0.313	0.755		*t* = 0.970	0.567
Male	509 (51.9)	49.60 (23.63)			87.02 (14.80)			27.42 (5.53)			27.12 (9.14)		
Female	472 (48.1)	48.32 (23.86)			87.01 (16.06)			27.31 (5.07)			26.79 (8.96)		
Grade			*F* = 1.121	0.326		*F* = 0.655	0.520		*F* = 0.059	0.943		*F* = 1.533	0.216
Seventh grade	300 (30.6)	50.06 (24.06)			90.88 (17.50)			27.29 (6.44)			26.54 (8.77)		
Eighth grade	349 (35.6)	47.47 (21.93)			90.78 (15.26)			27.30 (4.77)			26.83 (9.21)		
Ninth grade	332 (33.8)	49.59 (25.21)			89.55 (16.94)			27.43 (5.54)			27.75 (9.51)		
Annual income (in RMB)			*F* = 2.813	0.038^*^		*F* = 7.190	0.000^**^		*F* = 2.023	0.109		*F* = 4.179	0.006^**^
<10,000	162 (16.5)	53.88 (25.53)			88.87 (15.72)			28.09 (6.61)			28.39 (9.25)		
10,000–20,000	154 (15.7)	47.71 (25.56)			95.09 (18.96)			27.72 (6.02)			28.70 (9.38)		
>20,000	138 (14.1)	48.61 (21.98)			92.78 (18.15)			27.45 (5.12)			26.44 (10.48)		
Unclear	527 (53.7)	47.94 (22.92)			88.87 (15.24)			26.97 (5.18)			26.31 (8.65)		
Source of income			*F* = 0.742	0.527		*F* = 3.308	0.020^*^		*F* = 2.106	0.098		*F* = 1.688	0.168
Agriculture	49 (5.0)	48.02 (24.41)			89.56 (14.87)			28.04 (5.80)			27.37 (9.33)		
Business	81 (8.3)	45.40 (24.13)			95.70 (19.16)			28.63 (5.43)			29.03 (8.72)		
Out-migrant for work	768 (78.3)	49.44 (23.64)			90.10 (16.46)			27.22 (5.59)			26.93 (9.38)		
Others	83 (8.5)	48.77 (23.98)			88.46 (14.59)			26.77 (5.37)			25.99 (7.40)		
Only one child			*t* = 1.284	0.201		*t* = 0.442	0.659		*t* = 0.999	0.318		*t* = −1.751	0.082
Yes	132 (13.5)	51.80 (27.72)			87.62 (17.32)			27.80 (6.32)			25.48 (10.63)		
No	849 (86.5)	48.54 (23.04)			86.92 (15.10)			27.30 (5.13)			27.19 (8.76)		
Father goes out-migrant for work			*t* = 1.999	0.046		*t* = −0.471	0.638		*t* = 0.516	0.606		*t* = −1.511	0.131
Yes	896 (91.3)	49.45 (23.62)			86.84 (15.57)			27.39 (5.35)			26.82 (9.06)		
No	85 (8.7)	44.07 (24.47)			87.76 (13.74)			27.08 (4.89)			28.38 (8.86)		
Mother goes out-migrant for work			*t* = −0.229	0.819		*t* = −1.215	0.225		*t*=-2.280	0.023		*t* = −1.650	0.099
Yes	655 (66.8)	48.86 (23.21)			86.59 (14.99)			27.09 (4.98)			26.62 (8.75)		
No	326 (33.2)	49.23 (24.79)			87.86 (16.22)			27.91 (5.88)			27.63 (9.60)		
Father's working duration			*F* = 6.404	0.000^**^		*F* = 0.706	0.548		*F* = 0.354	0.787		*F* = 4.763	0.003^**^
<1 year	357 (36.4)	47.29 (23.15)			91.14 (16.48)			27.57 (4.86)			27.57 (9.12)		
2–5 years	137 (14.0)	46.08 (24.67)			90.96 (16.30)			27.16 (5.96)			28.25 (9.96)		
6–10 years	383 (39.0)	49.15 (23.51)			89.46 (16.85)			27.26 (6.18)			25.73 (8.95)		
>10 years	104 (10.6)	58.00 (23.44)			90.57 (15.90)			27.08 (5.06)			28.53 (8.73)		
Mother's working duration			*F* = 4.571	0.003^**^		*F* = 6.019	0.000^**^		*F* = 2.644	0.048^*^		*F* = 2.083	0.101
<1 year	496 (50.6)	48.42 (23.43)			90.95 (16.99)			27.73 (5.98)			27.32 (9.15)		
2–5 years	341 (34.8)	48.07 (23.82)			87.99 (15.50)			26.71 (5.07)			26.44 (9.12)		
6–10 years	75 (7.6)	47.55 (22.57)			96.27 (17.61)			27.96 (5.47)			26.11 (8.74)		
>10 years	69 (7.0)	59.09 (24.85)			91.89 (15.10)			27.00 (4.86)			29.13 (9.97)		
Father's educational level			*F* = 6.806	0.000^**^		*F* = 6.758	0.000^**^		*F* = 8.371	0.000^**^		*F* = 1.161	0.323
Below primary school	138 (14.1)	49.40 (23.36)			87.24 (15.72)			26.95 (5.22)			26.10 (9.68)		
Junior high school	591 (60.2)	50.77 (23.557)			89.61 (16.45)			26.90 (5.20)			26.97 (9.14)		
High school	212 (21.6)	42.73 (23.34)			94.57 (16.92)			29.01 (6.43)			27.60 (8.92)		
University or above	40 (4.1)	54.28 (24.59)			90.80 (14.67)			26.39 (5.58)			28.63 (9.43)		
Mother's educational level			*F* = 0.177	0.912		*F* = 4.698	0.003^**^		*F* = 4.433	0.004^**^		*F* = 0.545	0.651
Below primary school	161 (16.4)	49.99 (22.08)			86.92 (15.83)			26.44 (5.27)			27.79 (9.08)		
Junior high school	594 (60.6)	48.69 (23.37)			90.26 (16.59)			27.30 (5.20)			26.78 (9.16)		
High school	184 (18.8)	49.32 (26.24)			93.01 (16.29)			27.69 (5.44)			27.19 (9.45)		
University or above	42 (4.3)	47.74 (24.21)			94.14 (17.26)			29.81 (10.13)			27.38 (9.02)		
Father's occupation			*F* = 0.811	0.518		*F* = 1.710	0.146		*F* = 0.805	0.522		*F* = 1.580	0.177
Worker	521 (53.1)	48.73 (23.29)			90.49 (16.69)			27.39 (5.86)			27.04 (9.73)		
Farmer	133 (13.6)	51.24 (24.71)			88.79 (15.34)			26.84 (4.60)			27.21 (9.85)		
Public servant	27 (2.8)	51.11 (31.91)			90.78 (16.12)			26.19 (7.41)			27.70 (6.74)		
Businessman	114 (11.6)	50.34 (24.77)			93.71 (19.07)			27.83 (5.62)			28.61 (8.35)		
Others	186 (19.0)	46.93 (22.29)			89.21 (15.10)			27.43 (5.07)			25.91 (7.75)		
Mother's occupation			*F* = 1.203	0.308		*F* = 2.335	0.054		*F* = 1.198	0.310		*F* = 3.329	0.010^*^
Worker	337 (34.4)	47.49 (23.30)			91.07 (16.95)			27.33 (6.01)			26.90 (9.12)		
Farmer	197 (20.1)	48.06 (23.18)			90.59 (17.42)			27.57 (4.90)			28.67 (9.21)		
Public servant	95 (9.7)	50.07 (25.38)			91.17 (17.10)			26.59 (6.18)			24.57 (10.17)		
Business man	118 (12.0)	48.59 (23.37)			92.78 (16.99)			28.11 (5.84)			27.12 (7.98)		
Others	234 (23.9)	51.65 (24.27)			87.74 (14.35)			27.08 (5.04)			26.87 (9.24)		
Contact frequency			*F* = 0.481	0.750		*F* = 0.419	0.795					*F* = 2.770	0.026^*^
Every 3 days	211 (21.5)	48.48 (23.78)			90.91 (17.48)						26.81 (9.002)		
Every 1 week	275 (28.0)	49.39 (22.81)			89.79 (16.58)						26.77 (8.86)		
Every 2 weeks	47 (4.8)	47.17 (27.80)			92.90 (16.78)						24.18 (9.31)		
Every 3 weeks	131 (13.4)	47.05 (23.10)			90.25 (15.84)						26.26 (8.78)		
Every 1 month or above	317 (32.3)	50.02 (24.18)			90.27 (16.14)						28.21 (9.62)		

* *P < 0.05*,

** *P < 0.01*.

The analysis of variance results showed that the different annual income of families (*F* = 2.813, *P* = 0.038) had significant differences in life event scores, and further Student–Newman–Keuls pairwise comparisons showed no difference. There were significant differences in life events between fathers' different working duration (*F* = 6.404, *P* = 0.000). The life event scores in order, from high to low, were 6–10 years, <1 year, and 2–5 years. Similarly, there were significant differences in life events between mothers' different working duration (*F* = 4.571, *P* = 0.003). The life event scores in order, from high to low, were <1 year, 2–5 years, and 6–10 years.

### Descriptive Analysis of Life Events, Resilience, Self-Esteem, and Coping Style

The basic descriptive data for life events, resilience, self-esteem, and coping style are shown in Table 2. The mean total scores for life events were 49.98 ± 23.73 (range = 0–138), and the mean total scores for resilience were 87.01 ± 15.41 (range = 40–134), the mean total scores for self-esteem were 27.37 ± 5.31 (range = 10–60), and the mean total scores for coping style were 26.96 ± 9.05 (range = 0–58).

**Table 2:** Descriptive data for life events, resilience, self-esteem, and coping style (*N* = 981).

**Scale**	**No. of items**	**Possible range of scores**	**Actual range of scores**	**Mean**	**SD**
ASLEC	27	0–140	0–138	48.98	23.73
Interpersonal relationship	5	0–25	0–25	11.19	4.82
Study pressure	5	0–25	0–25	10.34	4.78
Punishment	7	0–35	0–35	10.93	7.57
Bereavement	3	0–15	0–15	4.97	4.20
Change for adaptation	4	0–20	0–20	6.35	3.83
Others	4	0–20	0–20	5.21	4.32
RSCA	27	27–135	40–134	87.01	15.41
Goal focus	5	5–25	5–25	17.36	4.21
Emotional control	6	6–30	6–56	19.02	5.21
Positive perception	4	4–20	4–44	14.33	3.55
Family support	6	6–30	5–66	16.63	4.42
Interpersonal assistance	6	6–30	6–30	19.67	5.86
RSES	10	10–60	10–60	27.37	5.31
SCSQ	20	0–60	0–58	26.96	9.05
Positive coping	12	0–36	0–36	18.24	6.70
Negative coping	8	0–24	0–24	8.72	4.61

*ASLEC, Adolescent self-rating life events checklist; RSCA, resilience scale Chinese adolescent; RSES, Rosenberg self-esteem scale; SCSQ, simplified coping style questionnaire*.

### Correlation Between Life Events, Resilience, Self-Esteem, and Coping Styles

Table 3 shows that each factor of life events was significantly negatively correlated with the total score of resilience and each dimension, self-esteem score, and total coping style scores. Additionally, the factors of interpersonal relationship, sense of loss, and healthy adaptation had significant negative correlation with positive coping style. Each factor of life events was significantly positively correlated with negative coping style; the factors of study pressure, punishment, and others had significant positive correlation with positive coping style.

**Table 3:** Correlation matrix for life events, resilience, self-esteem, and coping style.

	**1**	**2**	**3**	**4**	**5**	**6**	**7**	**8**	**9**	**10**	**11**	**12**	**13**	**14**	**15**	**16**	**17**	**18**
1	Interpersonal relationship	1																
2	Study pressure	0.58^**^	1															
3	Punishment	0.581^**^	0.658^**^	1														
4	Bereavement	0.327^**^	0.384^**^	0.478^**^	1													
5	Change for adaptation	0.49^**^	0.586^**^	0.602^**^	0.53^**^	1												
6	Others	0.508^**^	0.444^**^	0.706^**^	0.357^**^	0.512^**^	1											
7	TLE	0.752^**^	0.788^**^	0.898	0.648^**^	0.775^**^	0.764^**^	1										
8	Goal focus	−0.145^**^	−0.039	−0.086^**^	−0.081^**^	−0.058^**^	−0.187^**^	−0.127^**^	1									
9	Emotional control	−0.328^**^	−0.345^**^	−0.272^**^	−0.173^**^	−0.253^**^	−0.280^**^	−0.353^**^	0.287^**^	1								
10	Positive perception	−0.025	0.005	−0.074^**^	−0.074^**^	−0.083^**^	−0.108^**^	−0.076^**^	0.451^**^	0.126^**^	1							
11	Family support	−0.276^**^	−0.18^**^	−0.286^**^	−0.154^**^	−0.177^**^	−0.339^**^	−0.309^**^	0.342^**^	0.351^**^	0.178^**^	1						
12	Interpersonal assistance	−0.256^**^	−0.176^**^	−0.192^**^	−0.093^**^	−0.153^**^	−0.236^**^	−0.238^**^	0.330^**^	0.385^**^	0.167^**^	0.348^**^	1					
13	TR	−0.331^**^	−0.243^**^	−0.288^**^	−0.177^**^	−0.228^**^	−0.358^**^	−0.350^**^	0.685^**^	0.681^**^	0.508^**^	0.684^**^	0.729^**^	1				
14	SE	−0.276^**^	−0.286^**^	−0.271^**^	−0.182^**^	−0.256^**^	−0.280^**^	−0.332^**^	0.436^**^	0.409^**^	0.238^**^	0.402^**^	0.413^**^	0.579^**^	1			
15	Positive coping style	−0.035	0.063^**^	0.005	−0.003	0.051^**^	−0.071^*^	0.001	0.414^**^	0.156^**^	0.280^**^	0.247^**^	0.304^**^	0.413^**^	0.362^**^	1		
16	Negative coping style	0.194^**^	0.210^**^	0.285^**^	0.174^**^	0.244^**^	0.309^**^	0.306^**^	−0.095^**^	−0.261^**^	−0.052^**^	−0.182^**^	−0.206^**^	−0.255^**^	−0.190^**^	0.255^**^	1	
17	TCS	0.072^*^	0.153^**^	0.147^**^	0.085^**^	0.161^**^	0.103^**^	0.155^**^	0.261^**^	−0.016^**^	0.182^**^	0.092^**^	0.123^**^	0.179^**^	0.174^**^	0.873^**^	0.694^**^	1

*TLE, total life events; TR, total resilience; SE, self-esteem; TWC, total coping style*.

* *P < 0.05*,

** *P < 0.01*.

### Mediating Role of Resilience and Self-Esteem on Life Events and Coping Styles

First, the initial hypothetical model (Figure 1) showed unsatisfactory fit. In conforming to the modification indexes, we removed paths with low effects of the standardized path coefficient (<0.10). Therefore, after two revisions, we removed the path between self-esteem to negative coping style and self-esteem to positive coping style and increase the path between negative coping style to positive coping style in turn. The final model indicated a good fitting effect, as shown in Table 4. The χ^2^/*df* ratio was 4.994 (χ^2^ = 334.627, *df* = 67), and the RMSEA was 0.064. Furthermore, the CFI, GFI, NFI, RFI, and TLI values were higher than 0.900 (CFI = 0.949, GFI = 0.953, NFI = 0.938, RFI = 0.915, and TLI = 0.931).

**Table 4:** Correlation matrix for life events, resilience, self-esteem, and coping style.

**Model**	**χ^**2**^**	***df***	**χ^**2**^/*df***	**GFI**	**AGFI**	**CFI**	**NFI**	**RFI**	**IFI**	**TLI**	**RMSEA**
Hypothetical model	1379.313	143	9.646	0.871	0.828	0.783	0.765	0.783	0.784	0.796	0.094
Modified model	334.627	67	4.994	0.953	0.926	0.949	0.938	0.915	0.949	0.931	0.064

*GFI, goodness-of-fit index; NFI, normed fit index; IFI, incremental fit index; CFI, comparative of fit index; df, degree of freedom; RMSEA, root mean square error of approximation*.

**Figure 2:**
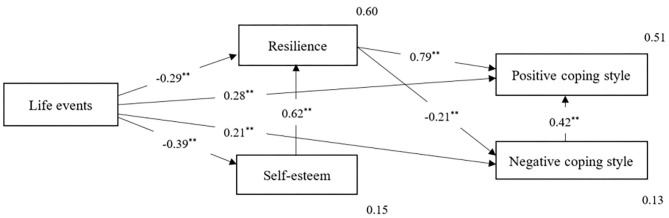
Standardized parameter estimates of the simplified model of effects on life events, positive coping way and negative coping way *via* resilience and self-esteem. *N* = 981. **P < 0.01.

Table 5 shows the total effect, standardized direct effect, and indirect effect of each variable; Table 6 displays the maximum likelihood estimate of the modified model. As shown in Figure 2, life events could negatively predict resilience (β = −0.29, *P* < 0.001) and self-esteem (β = −0.39, *P* < 0.001) and positively predict LBAs' positive coping style (β = 0.28, *P* < 0.001), and negative coping style (β = 0.21, *P* < 0.001). It indicated that the more serious the negative life events perceived by LBAs, the lower their own level of resilience and self-esteem, and the more they are likely to choose negative coping style, while positive coping style also exists. Further, self-esteem could positively predict LBAs' resilience (β = 0.62, *P* < 0.001), indicating that the higher the self-esteem level of LBAs, the higher their resilience. Moreover, resilience could negatively predict the negative coping style (β = −0.21, *P* < 0.001) and positively predict the positive coping style (β = 0.79, *P* < 0.001), indicating that the richer the resilience resources of the individual, the more they are able to avoid negative response, yet the more they tend to adopt a positive response.

**Table 5:** Standardized direct, indirect, and total effects for the modified model.

**Endogenous** **variables**	**Exogenous variables**	**Standardized direct effects**	**Standardized** **indirect effects**	**Standardized total effects**
Resilience	Life events	−0.286	−0.238	−0.524
	Self-esteem	0.618	0.000	0.618
Self-esteem	Life events	−0.385	0.000	−0.385
Positive coping	Life events	0.281	−0.279	0.002
	Negative coping	0.416	0.000	0.416
	Resilience	0.789	−0.086	0.703
Negative coping	Life events	0.214	0.108	0.322
	Resilience	−0.207	0.000	−0.207

**Table 6:** Maximum likelihood estimates of the modified model.

**Pathway**		**Non-standardized coefficients**	**Standardized** **coefficients**	**Standard errors**	**Critical ratio**	***P***
Self-esteem	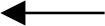 Life events	−0.621	−0.385	0.054	−11.410	0.000
Resilience	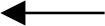 Life events	−0.201	−0.286	0.027	−7.552	0.000
Resilience	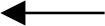 Self-esteem	0.269	0.618	0.020	13.302	0.000
Negative coping	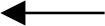 Life events	0.300	0.214	0.057	5.265	0.000
Negative coping	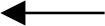 Resilience	−0.412	−0.207	0.090	−4.570	0.000
Positive coping	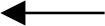 Resilience	2.287	0.789	0.173	13.252	0.000
Positive coping	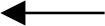 Life events	0.572	0.281	0.85	6.756	0.000
Positive coping	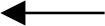 Negative coping	0.605	0.416	0.043	14.176	0.000

Obviously, resilience and self-esteem partially mediated the relationship between life events and coping styles. The direct effect value from life events to negative coping style was 0.21, and the total mediating effect was (−0.29) × (−0.21) + (−0.39) × 0.62 × (−0.21) = 0.11; the total effect was 0.32. The mediation effect amount was 34.37%, and the mediation effect amounts of the two indirect pathways were 19.03 and 15.86%, respectively.

## Discussion

In the current study, we employed SEM to investigate the relationships between life events, resilience, self-esteem, and coping styles among LBAs in China. Although some researchers have investigated some relationships between two or three variables (37–39, 51), few researches have explored the comprehensive relationships between four variables or the protective factors of coping styles, especially on dividing into positive and negative coping styles. Thus, the current study found that all the hypotheses we initially proposed have been supported, including the following: life events are negatively related to positive coping styles and positively related to negative coping styles; resilience mediates the relationship between life events and coping styles; and self-esteem mediates the relationship between life events and coping styles *via* resilience.

### Correlation Analysis of Life Events, Resilience, Self-Esteem and Coping Styles

Our research showed that life events of LBAs are significantly related to coping styles, which supported hypothesis 1. The factors of life events were significantly positively correlated with negative coping styles, which was consistent with Wang's research (37–39, 51). When LBAs encounter interpersonal relationships, learning pressure, and adapting to changes because of lack of timely help and support from parents, LBAs had no enough experience and ability to deal with these life events and might turn to adopt a negative coping style, which aggravated the negative impact of life events on LBAs.

Most studies (64–66) revealed that there is a negative correlation between life events and positive coping styles, whereas our study found that life events in general were positively correlated with positive coping style. Interpersonal relationships, sense of loss, and healthy adaptation have a significant negative correlation with positive coping styles. Faced with these major stress events, LBAs are less inclined to adopt positive coping styles and more inclined to deal with them with negative coping styles. This suggests that social workers should not only optimize the living environment of LBAs but also focus on reducing the frequency of life events, especially for interpersonal relationships, sense of loss, and healthy adaptation, and employ corresponding interventions for them to avoid adopt negative coping style. For another, our research found that learning pressure, punishment, and other factors are significantly positively correlated with positive coping styles. The Adaptive Calibration Model (67) believed that some kind of childhood adversities may enhance responsivity to the positive, supportive aspects of the environment; learning pressure and punishment may be such childhood adversities. When LBAs faced pressure from learning and punishment, they could accumulate experience and coping skills to respond to pressure positively. Therefore, appropriate learning pressure and punishment may promote psychological development and coping ability of LBAs, as well as adopt a positive coping.

### Mediating Role of Resilience and Self-Esteem on Life Events and Coping Styles

The current study showed that life events not only have direct effects on the negative coping style and positive coping style but also have indirect effects on coping styles by affecting resilience based on the SEM. This means that resilience mediated the relationship between life events and coping styles, which supported hypothesis 2. First, the study proved that life events, as an environmental factor that is not conducive to the normal development of individual psychology, have a significant negative effect on the mental health conditions of left-behind children, such as resilience. The longer the parent–child separation, the more frequent life events the LBAs may encounter, and the lower the level of mental health. Therefore, LBAs' parents can avoid working outside for a long time or increase the opportunities to come back home and communicate with children, and school can also hold various activities to enrich LBAs' lives and let them feel the love and support from teachers and classmates to reduce the negative impact of life events on the mental health of LBAs.

Therefore, results showed that those with a higher level of resilience tend to cope more positively with life events, which were consistent with the previous studies (68–70). Existing evidences (71, 72) suggested that resilience is an internal positive protective factor, which is manifested by a good adaptability to adverse life events. Meanwhile, resilience can be reflected by external factors such as attitudes or behaviors, including coping styles, and promote recovery of vulnerable groups from psychological crises and adopt positive coping styles under negative life events. Hence, resilience seemed to be one of the possible mechanisms to help LBAs cope with life events, which confirmed Kumpfer's resilience model that resilience is one of the most significant factors of healthy adaptation to stressful events. The life events (stressors) experienced by LBAs can play a role through their family support, interpersonal assistance, positive cognition, emotional control, and other psychological resilience factors (mediating protective factors) to reduce the adverse effects of life events on mental health (stress response) (31). Thus, LBAs with a higher level of resilience tended to adopt more positive coping styles such as seeking help from surroundings and focusing on problem-solving. Although LBAs have separated from their parents, and they are inevitable to encounter many life events, considering resilience could act as a buffer in the relationship between life events and coping styles, one of the most important approaches of coping with life events for LBAs is to enhance the levels of psychological resource reserve and resilience. Furthermore, some studies (73, 74) demonstrated that resilience-centric interventions are effective for developing positive cognition and coping with mental health problems in children and adolescents. This suggests that researchers could develop targeted resilience intervention programs for LBAs to improve their ability to positively cope with life events. For example (75), family-based parenting education and school-based peer support activities can be used for LBAs' resilience-building, which were proven to be effective.

Results also indicated that those with a higher level of self-esteem were more likely to cope with life events positively, and self-esteem cannot play the separate mediating role between life events and coping styles, relying on the mediating role of resilience, which were consistent with hypothesis 3. Self-esteem, as individuals' evaluation and perception of their own sense of life meaning and value, is considered to have an important role in maintaining mental health and promoting positive coping style (76). Previous studies (46, 77) also found that self-esteem is usually regarded as a protective factor for resilience, which can help adolescents improve their resilience level and resist failure and stress better. Some researchers (78) pointed out that self-esteem and resilience have important influence on individuals' cognition, emotions, and behaviors and may predict their coping styles. This indicated that adolescents with higher level of self-esteem usually show more confidence to cope with difficulties, including parents' absence or interpersonal relationship problems; are more likely to better manage their emotions; and take positive and confident ways to cope with life events, such as asking for help from teachers and classmates (79, 80), whereas those with low level of self-esteem tend to hold a negative perception of self-worth, have a low level of resilience, and perform negative coping styles to deal with difficulties, including self-blame, avoidance, and fantasy (81). Therefore, researchers should focus on the role of self-esteem to facilitate LBAs' positive coping style, especially attaching great importance to the mediating role of resilience in the relationship between self-esteem and coping styles, and develop targeted intervention programs to improve their levels of self-esteem. For instance, conduct lectures on mental health related to resilience and self-esteem, set up a psychological counseling room to provide targeted psychological counseling, and organize educational programs to cultivate resilience and self-esteem.

## Limitations

Although the study offered a preliminary conceptual framework of relationships between life events, resilience, self-esteem, and coping styles by using SEM, we acknowledged that the study has several limitations. First, the study used convenience sample to collect data, and the results could be biased toward those adolescents who were in good mental health conditions and motivated to share their perceptions. We still believe that it is unlikely that including the entire LBA population would have made much difference to the study results as the sample size was quite representative and the response rate was relatively high. Second, only LBAs in the rural junior high school in one province were selected in the current study. The sample may limit the generalizability of the results to wider age groups or geographical area. Future studies are recommended to include children in different stages of development and different locations in China or other countries. Third, although the researchers did their best to explain the questionnaire before collection, and participants were asked to finish questionnaires without the presence of teachers, our data were based on self-reports and may lead to information bias. Objective data collection method and data from parents, caregivers, teachers, and peers could complement our data. Fourth, although SEM is generally referred to produce information about causal relationships, the lack of use of longitudinal data prevents the interpretation form reflecting true causality. Therefore, further longitudinal or experimental studies should be conducted to better investigate causalities and the long-term effects of life events on coping styles of LBAs. Finally, the current study only focused on the relationships of life events, coping styles, resilience, and self-esteem among LBAs; future researchers could also identify the relationships between these psychological characteristics among NLBAs and compare the differences between LBAs and NLBAs.

## Conclusion

We found that all the hypotheses we proposed have been supported. Results indicated that life events negatively affected resilience and self-esteem and positively affected coping styles in LBAs, whereas resilience and self-esteem appeared to play a protective role. Meanwhile, results showed that resilience and self-esteem acted as mediators between life events and coping styles. Resilience can directly mediate life events and coping styles, whereas self-esteem mediates life events and coping styles *via* resilience. Our research provided preliminary insight into the mechanisms that have a significant influence on the relationship between life events, resilience, self-esteem, and coping styles among LBAs. The present study provides a basis for policy makers, educators, or practitioners to develop target school activities or intervention programs designed to promote LBAs to adopt positive coping style toward life events by enhancing their resilience and self-esteem.

## Data Availability Statement

The raw data supporting the conclusions of this article will be made available by the authors, without undue reservation.

## Ethics Statement

The study involving human participants were reviewed and approved by the ethics committee of Central South University (No: E201946). Written informed consent to participate in this study was provided by the participants' legal guardian/next of kin.

## Author Contributions

JZ is the primary investigator of the study and provided comments and ideas, and revised this paper. JL and Y-pC did data analysis, developed, and revised the manuscript. Y-fL provided comments and ideas, and helped revised the manuscript. S-lY, C-xT, Y-xT, and B-yY helped conduct the study, including developing survey, sampling, data analysis, and proof reading the manuscript. M-mL and MV provided substantial critical suggestions for manuscript revision, interpretation of data for the work, and revised the manuscript. All authors read and agreed on the final revision of the manuscript.

## Conflict of Interest

The authors declare that the research was conducted in the absence of any commercial or financial relationships that could be construed as a potential conflict of interest.
